# Use of Phenylephrine in Amlodipine Overdose With Refractory Hypotension and Noncardiogenic Pulmonary Edema: A Case Report

**DOI:** 10.7759/cureus.93024

**Published:** 2025-09-23

**Authors:** Samina Samina, Pawan Kumar, Kajal Bai

**Affiliations:** 1 Medicine, Jinnah Sindh Medical University, Karachi, PAK; 2 Medicine, Dow University of Health Sciences, Karachi, PAK; 3 Medicine, Chandka Medical College, Larkana, PAK

**Keywords:** amlodipine posioning, calcium, phenylephrine, pulmonary, refractory

## Abstract

Amlodipine is a commonly prescribed antihypertensive drug. We present a case of a young woman with a 70 mg amlodipine overdose admitted to our hospital with refractory hypotension and noncardiogenic pulmonary edema. She was initially managed in the intensive care unit (ICU) with intravenous sodium chloride 0.9% fluid boluses, calcium gluconate, and norepinephrine infusion, with poor response to therapy. After receiving phenylephrine 0.1 mg intravenous bolus, the patient showed improvement in blood pressure but experienced clinical worsening of pulmonary edema, which was subsequently managed with oxygen and furosemide therapy. With gradual clinical improvement, the patient was successfully discharged home on the fifth day. This case highlights that phenylephrine may be the drug of choice to treat refractory hypotension in amlodipine overdose without pulmonary edema.

## Introduction

Calcium channel blockers (CCBs) are commonly prescribed drugs for the management of hypertension and angina; however, they are also among the common causes of drug toxicity according to the surveillance system [1]. Amlodipine is a dihydropyridine calcium channel blocker that is widely used in medicine. Overdose of amlodipine may lead to refractory hypotension [2] and severe organ damage along with noncardiogenic pulmonary edema. Treatment of amlodipine overdose is mainly supportive and sometimes challenging [3]. Therapeutic options, including high-dose insulin, glucagon, or extracorporeal oxygen support, have shown benefit in certain cases, but with limitations due to the risk of serious side effects [3]. Vasopressors play a vital role in counteracting the effect of vasodilation caused by amlodipine, especially when started early. In this report, we share the case of a young patient who ingested 70 mg of amlodipine and developed refractory hypotension. With the use of phenylephrine along with supportive measures, the patient showed remarkable improvement. Our experience highlights and adds value to the existing literature on the potential role of phenylephrine in managing amlodipine overdose with refractory hypotension.

## Case presentation

A 31-year-old female with no known prior illnesses and psychiatric history presented to the emergency department within 24 hours after ingestion of 70 mg of amlodipine intentionally due to a conflict with her husband.

Initially, her husband noticed that she was complaining of vertigo, vomiting, and lethargy. She was taken to the hospital. In the emergency department, the initial vitals were as follows: blood pressure 80/40 mmHg, pulse 112 beats/min, temperature afebrile, respiratory rate 14 breaths/min, and oxygen saturation 95% on room air. On examination, she appeared comfortable, conscious, alert, and pale, and chest auscultation showed normal vesicular breathing. She was given intravenous fluids and shifted to the intensive care unit.

Initial laboratory investigations showed hemoglobin 9.5 mg/dL, total leukocyte count 15.5 × 10^9^/L, platelet count 455 × 10^9^/L, sodium 140 mmol/L, potassium 3.8 mmol/L, chloride 107 mmol/L, serum bicarbonate 21 mmol/L, blood urea 19 mg/dL, creatinine 0.7 mg/dL, and blood glucose 131 mg/dL (see Table 1 for laboratory values with reference ranges).

**Table 1:** Laboratory investigations with patient values and their corresponding reference ranges.

Investigation	Patient value	Reference range
Haemoglobin	9.5 g/dL	12–16 g/dL (female), 13–17 g/dL (male)
Total leukocyte count	15.5 x 10⁹/L	4–11 x 10⁹/L
Platelet count	455 x 10⁹/L	150–450 x 10⁹/L
Serum sodium	140 mmol/L	135–145 mmol/L
Serum potassium	3.8 mmol/L	3.5–5.0 mmol/L
Serum chloride	107 mmol/L	98–107 mmol/L
Serum bicarbonate	21 mmol/L	22–29 mmol/L
Blood urea	19 mg/dL	7–20 mg/dL
Serum creatinine	0.7 mg/dL	0.6–1.2 mg/dL
Blood glucose	131 mg/dL	70–140 mg/dL (random)

**Figure 1:**
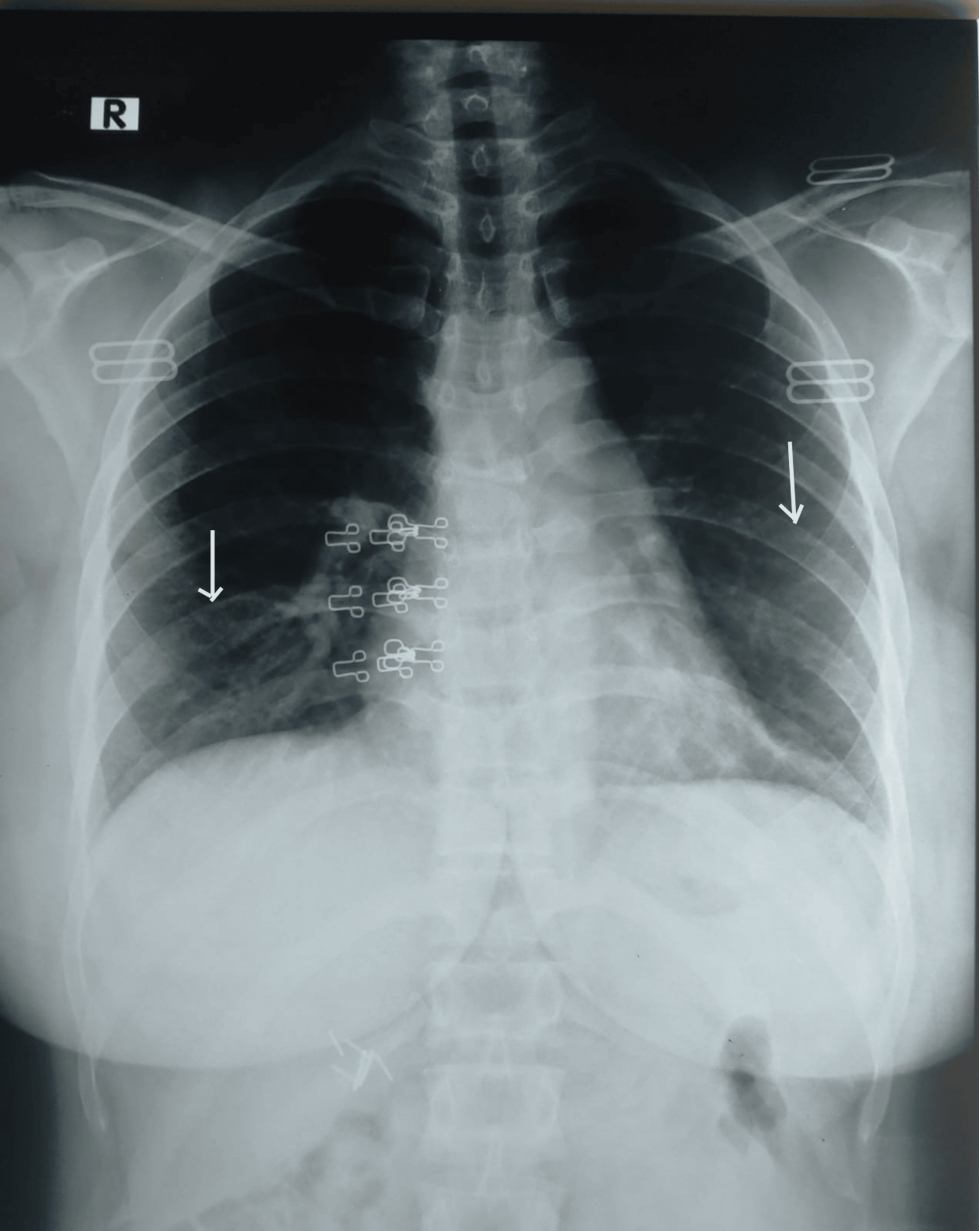
Chest X-ray (Day 1) with arrows showing bilateral mild interstitial infiltrates in the lower zones of the lung.

The electrocardiogram showed sinus tachycardia with no other abnormality. The initial chest X-ray (Figure 1) showed bilateral mild interstitial infiltrates in the lower zones. The 2D transthoracic echocardiography showed an ejection fraction of 55%, normal-sized cardiac chambers, normal left and right ventricular size, normal systolic function, and mild tricuspid regurgitation with PASP 25 mmHg.

She was given two liters of a fluid bolus of sodium chloride 0.9% injection and started on norepinephrine infusion (0.2 mcg/kg/min). She was also given calcium gluconate 2 g intravenously, but her blood pressure remained 90/40 mmHg. She was then given phenylephrine (intravenous 0.1 mg bolus), after which her blood pressure improved to 110/70 mmHg within 1 hour. Norepinephrine was tapered gradually over a 24-hour period and then stopped (see Table 2 for improvement in vitals following drug therapy).

**Table 2:** Patient's vitals chart showing changes in blood pressure and oxygen saturation with drug therapy.

Day	Therapy	Blood pressure (mmHg)	Pulse (beats/min)	Respiratory rate (breaths/min)	Oxygen saturation (%)
On Admission (Day 1)	IV sodium chloride 0.9% bolus (2 L)	80/40	112	14	95% on RA
Day 1 (before phenylephrine)	Inj. Norepinephrine 0.2 mcg/kg/min	92/46	115	14	95% on RA
Day 1 (after phenylephrine)	Inj. Phenylephrine 0.1 mg + Inj. Norepinephrine 0.2 mcg/kg/min	110/70	117	16	93% on RA
Day 2	Inj. Norepinephrine 0.01 mcg/kg/min	112/64	110	25	94% on 5 L O_2_
Day 3	No norepinephrine	107/82	105	24	95% on 2 L O_2_
Day 4	-	106/66	110	18	95% on 1 L O_2_
On Discharge (Day 5)	-	100/60	84	17	96% on RA

**Figure 2:**
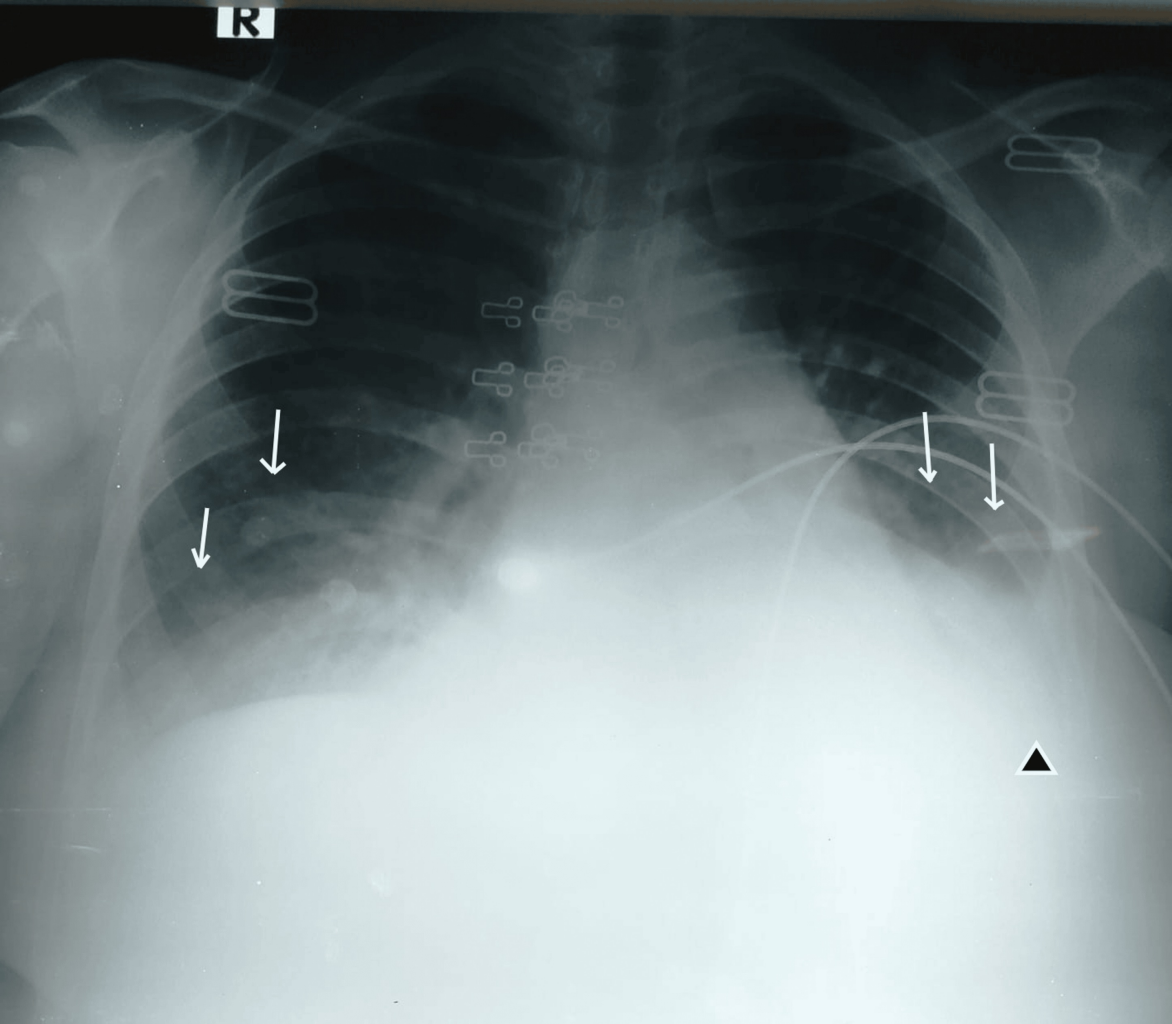
Chest X-ray (Day 2) with arrows showing worsening of bilateral alveolar and interstitial opacities. The arrow head shows left-sided pleural effusion with obliteration of costophrenic angle.

On the second day of admission, her oxygen saturation dropped to 90% on room air with mild respiratory distress. Chest auscultation showed inspiratory crackles. She was given oxygen at 5 L to keep saturation above 94%. Her repeated chest X-rays showed a bilateral increase in lower-zone alveolar and interstitial opacities along with mild left-sided pleural effusion (Figure 2). She was given furosemide, and an ultrasound-guided pleural tap was performed with 30 mL of clear yellow-colored fluid drained. The pleural fluid report showed total protein 3.0 g/dL, leukocyte count 77 cells/cmm (neutrophils 68%, lymphocytes 32%), RBC count 1000 cells/mm^3^, glucose 185 mg/dL, and LDH 149 IU/L.

On the third day, her oxygen demand gradually decreased, and she was shifted to the floor and discharged home on the fifth day.

## Discussion

Dihydropyridine CCBs relax vascular smooth muscles and reduce blood pressure with little impact on heart rate and contractility [4]. The half-life of amlodipine is 30-50 hours, and toxicity occurs when taken at more than 5-10 times the recommended dose [5]. The manifestations of amlodipine overdose include altered mental status, lethargy, hypotension, nausea, vomiting, metabolic acidosis, hyperglycemia, myocardial depression, and noncardiogenic pulmonary edema [6].

The hypotension in calcium channel blocker overdose is not due to hypovolemia but is due to vasodilation. Pulmonary edema and pleural effusion in amlodipine overdose are secondary to capillary leakage from systemic vasodilation.

Treatment generally includes supportive management. There is no specific antidote for amlodipine overdose [7]. Gastric lavage is beneficial if done within 1-2 hours of ingestion. Intravenous calcium may help reduce the competitive effects of calcium channel blockers; however, the response is sometimes inadequate [6]. When hypotension does not improve with intravenous calcium and vasoactive drugs, glucagon may be used due to its ability to increase cardiac contractility and improve blood pressure [5]; however, its use is limited by common side effects, including nausea, vomiting, ileus, and hypokalemia. Hyperinsulinemic-euglycemia is another recognized therapy that improves ionized calcium levels and increases the carbohydrate consumption of myocardial cells, thus improving cardiac contractility and blood pressure [8]. This high-dose insulin therapy is associated with hypoglycemia and hypokalemia, requiring strict blood glucose and potassium monitoring along with supplemental glucose and potassium administration.

We managed the refractory hypotension of our patient with phenylephrine 0.1 mg (intravenous bolus). After that, a remarkable improvement in blood pressure was observed, and norepinephrine infusion was tapered afterward. The therapeutic benefits of phenylephrine in our patient prevented multi-organ damage from refractory hypotension and reduced the length of intensive care unit stay.

Phenylephrine is a potent vasoconstrictor with alpha-1 agonist activity. However, its use is not without risks, as an excessive rise in afterload can lead to cardiac dysfunction with resultant heart failure, angina, pulmonary hypertension, and pulmonary edema [9]. The worsening of chest imaging and the increase in oxygen demand after the phenylephrine dose in our patient may have been related to these side effects of the therapeutic drug or may have been due to amlodipine toxicity. The complication was managed successfully with intravenous furosemide therapy and restriction of intravenous fluids.

## Conclusions

Amlodipine overdose management poses a significant therapeutic challenge. While supportive care remains the cornerstone of therapy, vasopressors play a critical role in counteracting the profound vasodilator effects. Our case highlights the potential role of phenylephrine as an effective option for managing refractory hypotension in amlodipine toxicity, particularly when used early. Nevertheless, its potential to worsen pulmonary edema in patients with compromised cardiac function or existing lung involvement should be carefully considered. More clinical experience and research are needed to better establish its role in the treatment of CCB toxicity.
